# Mice deleted for cell division cycle 73 gene develop parathyroid and uterine tumours: model for the hyperparathyroidism-jaw tumour syndrome

**DOI:** 10.1038/onc.2017.43

**Published:** 2017-03-13

**Authors:** G V Walls, M Stevenson, K E Lines, P J Newey, A A C Reed, M R Bowl, J Jeyabalan, B Harding, K J Bradley, S Manek, J Chen, P Wang, B O Williams, B T Teh, R V Thakker

**Affiliations:** 1 Academic Endocrine Unit, Oxford Centre for Diabetes, Endocrinology and Metabolism (OCDEM), Radcliffe Department of Medicine, University of Oxford, Churchill Hospital, Oxford, UK; 2 Department of Pathology, John Radcliffe Hospital, Headley Way, Oxford, UK; 3 Laboratory of Cancer Genetics, Van Andel Research Institute, Grand Rapids, MI, USA; 4 Center for Cancer and Cell Biology, Van Andel Research Institute, Grand Rapids, MI, USA

## Abstract

The hyperparathyroidism-jaw tumour (HPT-JT) syndrome is an autosomal dominant disorder characterized by occurrence of parathyroid tumours, often atypical adenomas and carcinomas, ossifying jaw fibromas, renal tumours and uterine benign and malignant neoplasms. HPT-JT is caused by mutations of the cell division cycle 73 (*CDC73*) gene, located on chromosome 1q31.2 and encodes a 531 amino acid protein, parafibromin. To facilitate *in vivo* studies of Cdc73 in tumourigenesis we generated conventional (*Cdc73*^+/−^) and conditional parathyroid-specific (*Cdc73*^+/L^/*PTH-Cre* and *Cdc73*^*L/L*^/*PTH-Cre*) mouse models. Mice were aged to 18-21 months and studied for survival, tumour development and proliferation, and serum biochemistry, and compared to age-matched wild-type (*Cdc73*^+/+^ and *Cdc73*^+/+^*/PTH-Cre*) littermates. Survival of *Cdc73*^+/−^ mice, when compared to *Cdc73*^+/+^ mice was reduced (*Cdc73*^+/−^=80% *Cdc73*^+/+^=90% at 18 months of age, *P*<0.05). *Cdc73*^+/−^*, Cdc73*^+/L^*/PTH-Cre* and *Cdc73*^*L/L*^*/PTH-Cre* mice developed parathyroid tumours, which had nuclear pleomorphism, fibrous septation and increased galectin-3 expression, consistent with atypical parathyroid adenomas, from 9 months of age. Parathyroid tumours in *Cdc73*^+/−^*, Cdc73*^+/L^*/PTH-Cre* and *Cdc73*^*L/L*^*/PTH-Cre* mice had significantly increased proliferation, with rates >fourfold higher than that in parathyroid glands of wild-type littermates (*P*<0.0001). *Cdc73*^+/−^*, Cdc73*^+/L^*/PTH-Cre* and *Cdc73*^*L/L*^*/PTH-Cre* mice had higher mean serum calcium concentrations than wild-type littermates, and *Cdc73*^+/−^ mice also had increased mean serum parathyroid hormone (PTH) concentrations. Parathyroid tumour development, and elevations in serum calcium and PTH, were similar in males and females. *Cdc73*^+/−^ mice did not develop bone or renal tumours but female *Cdc73*^+/−^ mice, at 18 months of age, had uterine neoplasms comprising squamous metaplasia, adenofibroma and adenomyoma. Uterine neoplasms, myometria and jaw bones of *Cdc73*^+/−^ mice had increased proliferation rates that were 2-fold higher than in *Cdc73*^+/+^ mice (*P*<0.05). Thus, our studies, which have established mouse models for parathyroid tumours and uterine neoplasms that develop in the HPT-JT syndrome, provide *in vivo* models for future studies of these tumours.

## Introduction

Mutations of the cell division cycle 73 (*CDC73*) gene (OMIM #607393), which is located on chromosome 1q31.2 and encodes a 531 amino acid protein called parafibromin, are associated with hereditary and non-hereditary forms of parathyroid carcinomas and the hyperparathyroidism-jaw tumour (HPT-JT) syndrome (OMIM #145001).^1, 2, 3^ HPT-JT, an autosomal dominant disorder, is characterized by the occurrence of parathyroid tumours, and ossifying fibromas of the jaw which occur in ~30% of HPT-JT patients (Table 1).^1, 4, 5, 6^ The parathyroid tumours are usually parathyroid adenomas (PAs) but may be atypical parathyroid adenomas (APAs) or parathyroid carcinomas (PCs) in >15% of HPT-JT patients. In addition, >15% of HPT-JT patients may also develop renal tumours, which include Wilms’ tumours, hamartomas and carcinomas, and ~75% of women with HPT-JT will develop, at an early age, benign and malignant neoplasms of the uterus that may be associated with recurrent miscarriages and severe menorrhagia, requiring hysterectomy.^4, 5, 7, 8, 9^ These uterine neoplasms which all arise in cells derived from the embryonic mesodermal Mullerian duct system, comprise extensive adenomyosis, adenofibromas, endometrial hyperplasia, leiomyosis and adenosarcomas in ~55%, ~35%, ~30%, ~30% and ~15% of patients, respectively, and women with HPT-JT, may have more than one type of uterine neoplasm (Table 1). Other tumours that may arise in <2% of HPT-JT patients are Hürthle cell thyroid adenomas, pancreatic adenocarcinomas and mixed germ cell testicular tumours.^4, 5, 7, 8^ *CDC73* loss of heterozygosity (LOH) has been observed in HPT-JT associated tumours, thereby indicating a likely tumour suppressor role for *CDC73.*^1^ A tumour suppressor role for *CDC73* is further supported by reports that the majority of *CDC73* mutations are predicted to result in a functional loss of parafibromin, and that some HPT-JT tumours and non-hereditary PCs harbour both germline and somatic mutations, consistent with the Knudson 'two-hit' hypothesis.^10, 11, 12^

**Figure 1:**
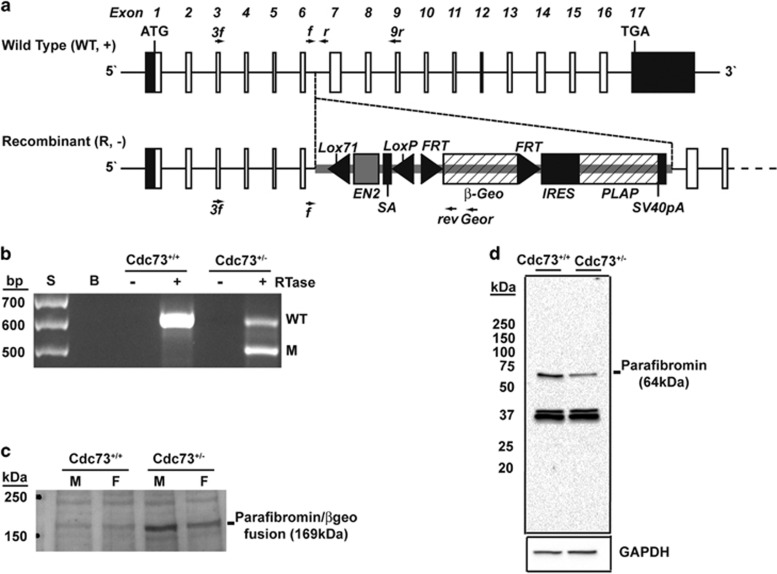
Establishing a conventional *Cdc73* knockout mouse model. (**a**) Schematic diagram of the *Cdc73* gene representing the wild-type (WT, +) and recombinant (R, −) alleles. ATG and TGA represent the start and stop codons respectively. The exons are represented by boxes (open boxes depict translated regions), and the domains within the GeneTrap (Gt) vector, incorporated into the R allele, are labelled. The GeneTrap vector in the RRE190 ES cells from BayGenomics^22^ referred to as Gt^(RRE190Byg)^ is inserted into intron 6 of the *Cdc73* gene and subsumes normal splicing of the *Cdc73* exon 6 donor site to the GeneTrap Engrailed2 (EN2) acceptor site with loss of exons 7–17. Thus, the parafibromin-β-geo fusion from the first six exons of the *Cdc73* gene, would contain only the N-terminal 170 amino acids, which encompasses the nuclear localization signal, and lack the remaining 361 amino acids which will encompass the domains that interact with the Paf1 complex, histone methyltransferase complexes,^16^ and β-catenin,^17^ of the wild-type parafibromin. A loss of these critical domains would render the expressed mutant parafibromin non-functional. *LoxP* (locus of crossing over in phage *P1*)*, Lox71* (locus of crossing over 71)*, FRT* (flippase recognition target)*, SA* (splice acceptor of mouse)*, βGeo* (EN2 exon 2, fusion of β-galactosidase and neomycin transferase)*, IRES* (internal ribosome entry site)*, PLAP (*placental alkaline phosphatase) and *SV40pA* (Simian virus 40 polyadenylation signal). The Lox71, LoxP and FRT sites, located in the GeneTrap vector allow the capability to remove the engrailed intron and β-geo, by breeding with Cre or Flp expressing mice, in order to restore gene function. However, this was not undertaken for this study. (**b**) Identification of wild-type (*Cdc73*^+/+^) and heterozygous (*Cdc73*^+/−^) mice by RT–PCR using template RNA extracted from kidneys of adult mice and primers (3 f, 9r and rev). The sizes of the wild-type (WT) and mutant (M) bands are 593 and 500 bp, respectively. (**c**) Western blot analysis of kidney lysates from adult *Cdc73*^+/+^ and *Cdc73*^+/−^ mice, utilizing an anti-β-geo antibody, revealed the expression of a parafibromin/beta-geo fusion protein (169 kDa) in *Cdc73*^+/−^ knockout mice only. (**d**) Analysis of parafibromin expression (64 kDa) using an anti-parafibromin antibody (A300-171A) revealed a 50% reduction in expression assessed by densitometry of band intensity normalized for GAPDH expression (*n*=4) in *Cdc73*^+/−^ mice compared to *Cdc73*^+/+^ mice; the whole Western blot is shown. The specificity of the anti-parafibromin antibody was validated in HeLa cells transfected with siRNA against *CDC73* (Supplementary Figure 1). These results also show that only one form of parafibromin of size 64 kDa and representing the 531 amino acid protein is expressed by *Cdc73*, despite the reports of of 6 *Cdc73* transcripts in the Ensembl database.^75^ These 6 murine *Cdc73* transcripts comprise: transcript 1 which is 11586 bp and encodes a 531 amino acid protein; transcripts 2, 4, 5 and 6 which are 3134 bp, 3056 bp, 2727 bp and 1047 bp, respectively, in length and are processed transcripts or retained introns that do not lead to protein products; and transcript 3 that is 479 bp in length and results in a 73 amino acid protein and subject to nonsense mediated decay. Thus, the observation of only one form of parafibromin of 64 kDa, is consistent with the translation of transcript 1 in the Ensembl database,^75^ which results in the 531 amino acid parafibromin. The 37 kDa bands, which do not correspond to any translated proteins from the other transcripts, are present with similar intensities in *Cdc73*^+/−^ and *Cdc73*^+/+^ mice (*P*=0.493, *n*=4), and are likely to represent non-specific bands. Such non-specific bands, which were not altered by the use of CDC73 siRNA (siCDC73), were also detected in HeLa cells (Supplementary Figure 1). B, blank; F, female; M, male; RTase, reverse transcriptase; S, size marker.

Parafibromin, which is encoded by exons 1–17 of *CDC73* (Figure 1a), is a ubiquitously expressed predominantly nuclear protein that is evolutionary conserved.^1^ Moreover, the ~200 amino acids of the terminal segment of parafibromin have ~27% sequence identity and 47% similarity to the yeast Cdc73 protein, which is a component of the polymerase-associated factor-1 (Paf1) complex,^13^ a key transcriptional regulatory complex that interacts directly with RNA polymerase II. The crystal structure of the yeast Cdc73 C-domain has been reported to adopt a Ras-like fold that participates in histone ubiquitination and methylation steps through both promoter and coding regions, and studies have shown that human homologues of the yeast Paf1 complex are associated with parafibromin.^14, 15, 16^ Moreover, parafibromin and its *Drosophila* homologue, Hyrax, which is a component of the Wnt1 wingless pathway and has an essential role in normal embryonic development, have a high degree of sequence similarity in their C-terminal portions.^17, 18^ This suggested that parafibromin may have a role in embryonic development and studies of mice deleted for *Cdc73* have also shown that parafibromin has key roles in mammalian embryonic development.^19^ Thus, *Cdc73* null mice were embryonic lethal by 6.5 day post-coitum, which is the stage when implantation occurs.^19^ Parafibromin and Hyrax also have a high degree of sequence similarity in their N-terminal domains, which directly interact with β-catenin/Armadillo in the context of the PAF1 complex.^17^ The role of parafibromin as a mediator of Wnt signalling is supported by studies in human HEK293 cells, which has shown that Wnt target gene expression is directly correlated with parafibromin expression.^17^ Moreover, parafibromin overexpression in HEK293 and NIH3T3 cells strongly inhibits proliferation, and in HeLa cells it increases G1 phase arrest and apoptosis with a concomitant reduction in S-phase entry, and a resulting downregulation of the cell cycle regulator cyclin D1, which is an oncogene known to be upregulated in parathyroid tumours.^19, 20, 21^ Furthermore, underexpression of parafibromin, induced by RNAi, has been reported to increase the proportion of HeLa cells in S-phase, and to reduce basal apoptosis.^13^ These *in vitro* findings indicate that parafibromin is a likely tumour suppressor in mammalian cells, and to explore further the role of parafibromin as an *in vivo* tumour suppressor we studied mice deleted for *Cdc73* for the development of tumours.

## Results

### Generation, viability and survival of mice deleted for Cdc73 alleles

**Figure 2:**
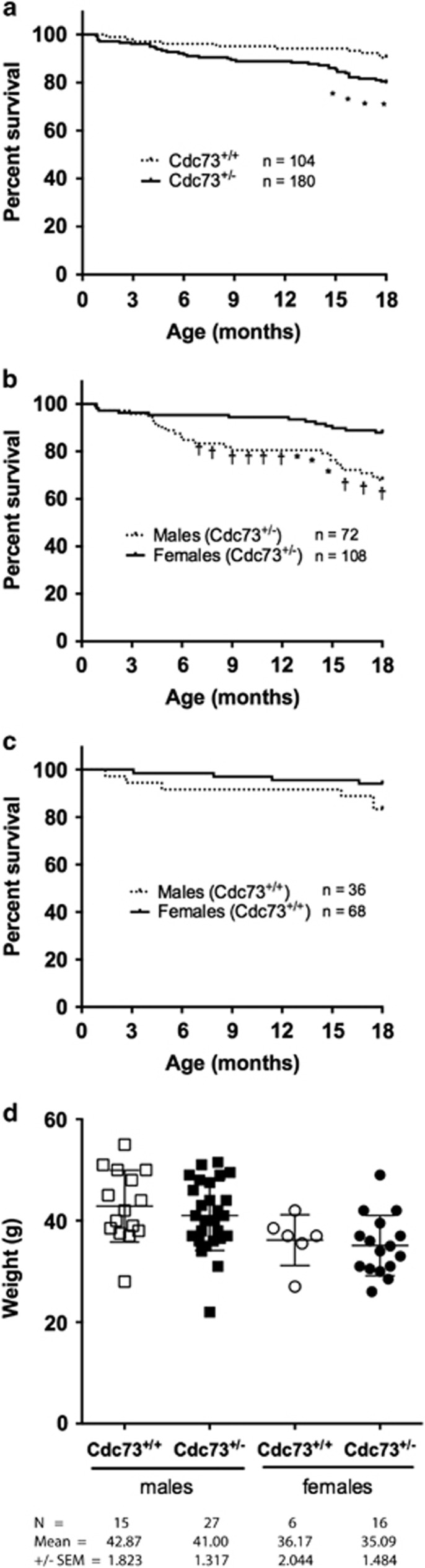
Survival and weights of *Cdc73*^+/+^ and conventional *Cdc73*^+/−^ knockout mice. (**a**) Survival of *Cdc73*^+/+^and *Cdc73*^+/−^ mice (data for male and female mice combined) over 18 months. Kaplan–Meier analysis revealed significantly lower survival in *Cdc73*^+/−^ mice when compared to *Cdc73*^+/+^ mice (80 versus 90%, Log-rank Mantel Cox test, *P*<0.05). Moreover the reduced survival in the *Cdc73*^+/−^ mice was observed from 15 months of age (*P<0.05, Fisher’s exact test). (**b**) Kaplan–Meier analysis showed that *Cdc73*^+/−^ males had significantly reduced survival than *Cdc73*^+/−^ females (Log-rank Mantel Cox test, *P*<0.005), which was observed from the age of 7 months (*P<0.05, ^†^*P*<0.01, Fisher’s exact test). (**c**) Kaplan–Meier analysis showing that survival of male and female *Cdc73*^+/+^ mice was similar. Moreover, the survival of *Cdc73*^+/+^ male mice did not differ significantly (*P*=0.094) from that of *Cdc73*^+/−^ male mice (see above). (**d**) Bodyweights at 18 months of age of *Cdc73*^+/+^and *Cdc73*^+/−^ mice, showing that the mean bodyweights of *Cdc73*^+/+^and *Cdc73*^+/−^ males, and of *Cdc73*^+/+^and *Cdc73*^+/−^ females were similar.

Conventional *Cdc73* knockout mice were established using the embryonic stem (ES) cell line (RRE190) from Bay Genomics Genetrap resource (Figure 1a),^22^ as described,^19^ and congenic animals obtained by backcrossing onto wild-type C57BL/6 females for ten generations. Expression of wild-type and mutant *Cdc73* and parafibromin, was detected by RT-PCR (Figure 1b) and western blot analysis (Figure 1c), respectively, to establish the wild-type (*Cdc73*^+/+^) and heterozygote (*Cdc73*^*+/Gt(RRE190)Byg*^, referred to as *Cdc73*^+/−^) genotypes of adult mice. *Cdc73*^+/−^ mice were viable and fertile and homozygote (*Cdc73*^*Gt(RRE190)Byg/Gt(RRE190)Byg*^, referred to as *Cdc73*^*-/-*^) mice have been previously reported to demonstrate embryonic lethality.^19^ Kaplan–Meier analysis of 284 mice, comprising 104 *Cdc73*^+/+^ mice (*n*=36 male, 68 female) and 180 *Cdc73*^+/−^ mice (*n*=72 male, 108 female) aged to 18 months, revealed a significantly decreased survival of *Cdc73*^+/−^ mice compared to *Cdc73*^+/+^ mice (survival of *Cdc73*^+/−^ versus *Cdc73*^+/+^ mice=80% versus 90%, Figure 2a, *P*<0.05). Further analysis of this data by gender, revealed that the decreased survival of *Cdc73*^+/−^ mice was largely due to decreased survival of male *Cdc73*^+/−^ mice, which was observed from 7 months of age (Figure 2b); the survival of *Cdc73*^+/+^ male and female mice was similar (Figure 2c). The decreased survival in male *Cdc73*^+/−^ mice was not associated with lower bodyweight (Figure 2d), which was consistent with the reported mean bodyweight for C57BL/6 mice of similar ages.^23^

Parathyroid-specific *Cdc73* conditional knockout mice, were generated by mating parathyroid hormone (PTH)-Cre transgenic mice^24^ with previously established *Cdc73*-floxed mice (*Cdc73*^*L/L*^).^19^ This resulted in mice deleted for one or both *Cdc73* alleles in the parathyroids, that yielded heterozygote *Cdc73*^+/L^/*PTH-Cre* and homozygote *Cdc73*^*L/L*^/*PTH-Cre* mice, respectively,^19, 24^ which were viable and fertile. A total of 52 parathyroid-specific *Cdc73* knockout mice were generated, and comprised 20 *Cdc73*^+/+^*/PTH-Cre* mice, 15 *Cdc73*^+/L^*/PTH-Cre* mice and 17 *Cdc73*^*L/L*^*/PTH-Cre* mice.

### Examination for development of HPT-JT associated tumours.

Development of HPT-JT associated tumours was assessed in 69 mice (21 *Cdc73*^+/+^ mice (9 males and 12 females) and 48 *Cdc73*^+/−^ conventional knockout mice (12 males and 36 females)) between the ages of >7 to <24 months (Table 1). Parathyroid tumours were found to occur in 68% of *Cdc73*^+/−^ mice, and 25% of these were adenomas and 75% were APAs (defined by having collagenous fibrous septa,^25^ and/or immunostaining for galectin-3 but lacking evidence of invasion or metastasis). We used immunostaining for galectin-3, which is an anti-apoptotic lectin that regulates cyclin D1 and C-Jun N-terminal kinase 1 (*JNK1*) expression and promotes tumour growth and metastasis,^26, 27^ as it has been reported to have a sensitivity of >95% and specificity of 90% for pathological diagnosis of PC in man.^28, 29^ Uterine neoplasms were found in ~33% of *Cdc73*^+/−^ females (Table 1). Parathyroid tumours or uterine neoplasms were not found to occur in *Cdc73*^+/+^ mice. Ossifying fibromas of the jaw, and tumours of the kidneys, thyroid, pancreas or testis (Table 1) were not found in any of the *Cdc73*^+/−^ or *Cdc73*^+/+^ mice. Parathyroid tumour development was also found to occur in >40% of the 32 conditional knockout mice, which comprised 15 *Cdc73*^+/L^*/PTH-Cre* mice (9 males and 6 females) and 17 *Cdc73*^*L/L*^*/PTH-Cre* mice (8 males and 9 females), but not in any of 20 *Cdc73*^+/+^*/PTH-Cre* mice (11 males and 9 females), aged 20–21 months of age. The parathyroid and uterine neoplasms developing in the mutant mice were further studied.

### Analysis of parathyroid tumours

**Figure 3:**
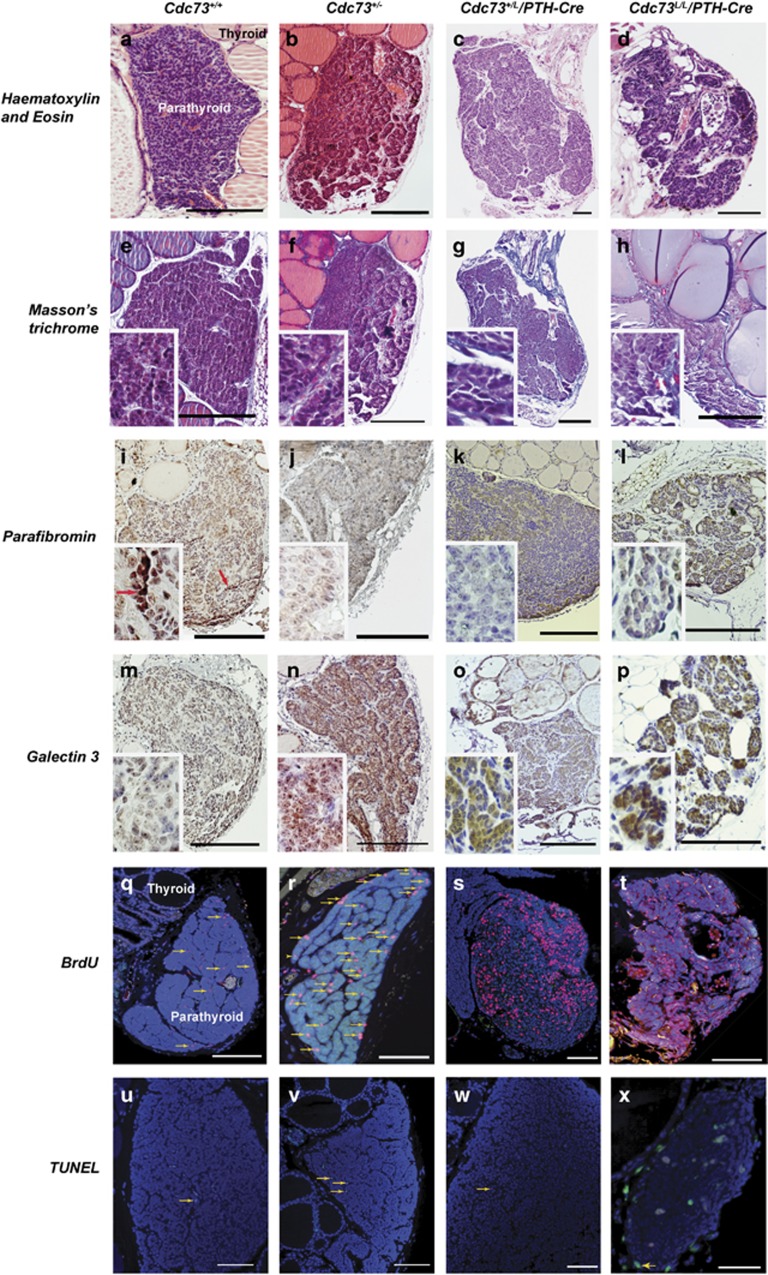
Parathyroid tumours develop in *Cdc73*^+/−^ and parathyroid-specific *Cdc73*^+/L^/*PTH-Cre* and *Cdc73*^*L/L*^/*PTH-Cre* knockout mice. (**a**–**d**) H&E-stained sections of parathyroid glands from wild-type (*Cdc73*^+/+^), heterozygote (*Cdc73*^+/−^ and *Cdc73*^+/L^/*PTH-Cre*), and homozygote (*Cdc73*^*L/L*^/*PTH-Cre*) mice, showing: (**a**) homogenous histology of a wild-type parathyroid; (**b**) enlarged PA from a *Cdc73*^+/−^ mouse; (**c**) a large PA from a *Cdc73*^+/L^/*PTH-Cre* mouse, with increased septation and irregular outline; and (**d**) abnormal architecture of a parathyroid gland from a *Cdc73*^*L/L*^/*PTH-Cre* mouse, with increased lipid deposition, nodularity, necrosis and septation. (E-H) Masson’s trichrome stained sections of parathyroids of each genotype demonstrating collagen (blue), and increased fibrous septation in *Cdc73*^+/−^, *Cdc73*^+/L^/*PTH-Cre* and *Cdc73*^*L/L*^/*PTH-Cre* mice when compared to *Cdc73*^+/+^ mice. (**i**–**l**) Nuclear parafibromin protein expression (brown) in parathyroids was absent or reduced in *Cdc73*^+/−^, *Cdc73*^+/L^/*PTH-Cre* and *Cdc73*^*L/L*^/*PTH-Cre* mice (**j**–**l**), when compared to *Cdc73*^+/+^ mice (**i**) (a cluster of parafibromin expressing cells is indicated by the red arrow in panel and inset). Importantly, nuclear parafibromin expression was not reduced in pancreatic exocrine and exocrine cells, endothelial cells and thyroid epithelial cells of *Cdc73*^+/L^/*PTH-Cre* and *Cdc73*^*L/L*^/*PTH-Cre* mice, thereby confirming the parathyroid-specific loss of *Cdc73* expression resulting from the presence of PTH-Cre (Supplementary Figure 3). (**m**–**p**) Galectin-3 protein expression (brown cytoplasm) in parathyroids was increased in *Cdc73*^+/−^, *Cdc73*^+/L^/*PTH-Cre* and *Cdc73*^*L/L*^/*PTH-Cre* mice (**n–p**), when compared to *Cdc73*^+/+^ mice (**m**). (**q**–**t**) Assessment of parathyroid tumour proliferation by immunofluorescent BrdU incorporation, by continuous administration of BrdU in drinking water, showing that: (**q**) few parathyroid cells had proliferated in *Cdc73*^+/+^ mice; but that (**r**–**t**) higher proportions of parathyroid cell nuclei had incorporated BrdU in the parathyroid tumours of *Cdc73*^+/−^, *Cdc73*^+/L^/*PTH-Cre* and *Cdc73*^*L/L*^/*PTH-Cre* mice. (**s**) A rim of normal parathyroid tissue, to the left of the image, had low proliferation, whilst the tumour nodule demonstrated focal areas with a high proportion of nuclei that had incorporated BrdU. BrdU-containing nuclei (red, arrows) indicate cellular proliferation; DAPI nuclear counterstain (blue). (**u**–**x**) Assessment of apoptosis by TUNEL assay. Apoptotic cells (green nuclei, arrows) were infrequently observed in parathyroids from mice of each genotype; DAPI nuclear counterstain (blue). Scale bars represent 200 μm; insets have x400 magnification.

Parathyroid glands were identified in 74% of mice (*n*=109) and the remaining 26% of mice in whom parathyroid glands were not identified were evenly distributed among all the genotypes. Parathyroid tumours were found in >65% of *Cdc73*^+/−^ mice (*n*=15/22) but in none of 16 *Cdc73*^+/+^ littermates (*P*<0.0001, two-tailed Fisher’s exact test, Table 1), between 9 and 18 months of age; and in ⩾50% of ⩾18-month-old *Cdc73*^+/L^/*PTH-Cre* (*n*=7/12) and *Cdc73*^*L/L*^/*PTH-Cre* (*n*=6/12) mice, but not in any of 19 *Cdc73*^+/+^/*PTH-Cre* littermates (*P*<0.005). The parathyroids in wild-type mice were ~500 μm in length (Supplementary Figure 2) and had a homogenous appearance (Figure 3a). Parathyroid tumours (Figures 3b–d), which were ~1 mm in length (Supplementary Figure 2) and had a heterogenous architecture, developed in *Cdc73*^+/−^, and *Cdc73*^+/L^*/PTH-Cre* and *Cdc73*^*L/L*^*/PTH-Cre* mice, and these demonstrated abnormalities that included glandular enlargement, nuclear pleomorphism, and septation (Figures 3b–d), which are features often observed in PCs and APAs. Indeed 75% of the parathyroid tumours of *Cdc73*^+/−^, *Cdc73*^+/L^/*PTH-Cre* and *Cdc73*^*L/L*^/*PTH-Cre* mice, when compared to wild-type littermates had features found in APAs (Table 1),^1, 9^ that included: increased collagen deposition in the septa (Figures 3e–h); reduced nuclear expression of parafibromin (Figures 3i–l); and increased expression of galectin-3 (Figures 3m–p). Loss of retinoblastoma protein expression and increased cyclin D1 expression, which are found in >95% and 90%^30^ of human PCs, respectively, were not found to occur in the parathyroid tumours from *Cdc73*^+/−^, *Cdc73*^+/L^/*PTH-Cre* and *Cdc73*^*L/L*^/*PTH-Cre* mice (data not shown). To assess the proliferation rate of these parathyroid tumours, mice were given the thymidine analogue BrdU in drinking water for two weeks,^31^ and the proportion of cells that had incorporated nuclear BrdU was calculated (Figures 3q–t, Table 2). The parathyroid tumours developing in the *Cdc73*^+/−^ and *Cdc73*^+/L^/*PTH-Cre* mice had significantly higher daily proliferation rates, by three- to fourfold, while that of *Cdc73*^*L/L*^/*PTH-Cre* mice was ~9-fold higher, when compared to those of parathyroid glands in wild-type mice *Cdc73*^+/+^ and *Cdc73*^+/+^/*PTH-Cre* mice (Table 2, *P*<0.0001). Apoptotic rates in parathyroids of *Cdc73*^+/−^, *Cdc73*^+/L^/*PTH-Cre* and *Cdc73*^*L/L*^/*PTH-Cre* mice were not significantly different from wild-type (*Cdc73*^+/+^ and *Cdc73*^+/+^/*PTH-Cre*) littermates (Figures 3u–x, Supplementary Figure 4).

**Figure 4:**
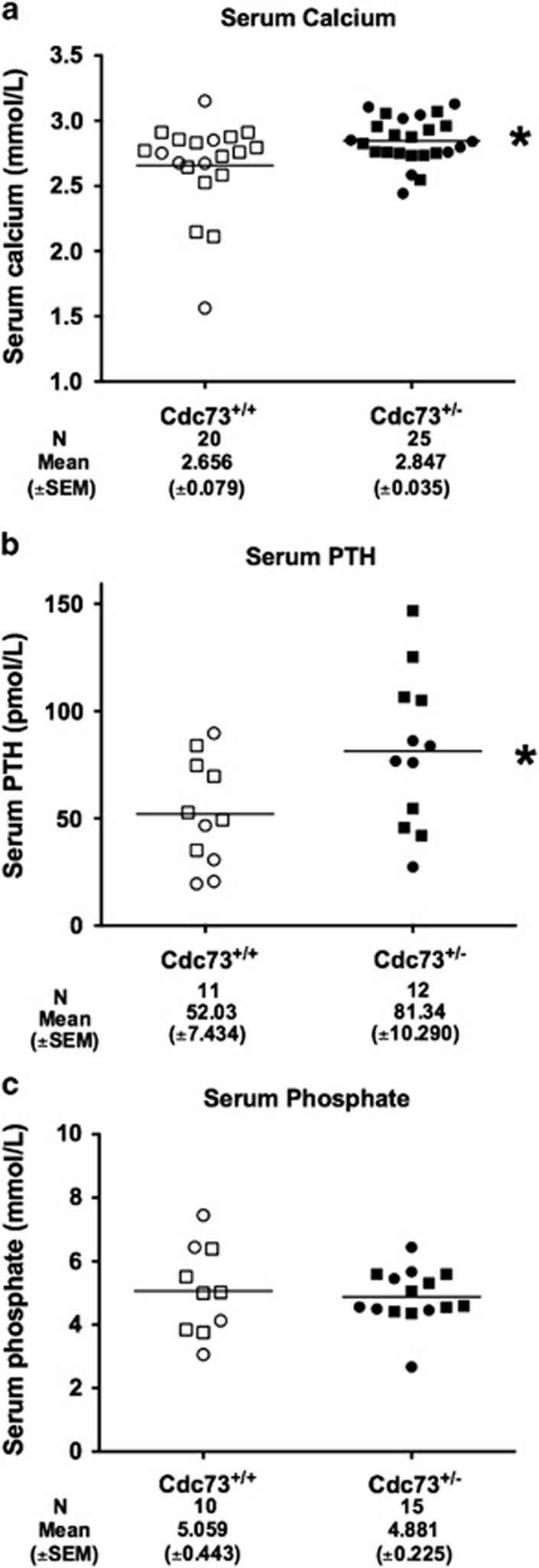
*Cdc73*^+/−^ mice have increased mean serum calcium and PTH concentrations, when compared to *Cdc73*^+/+^ mice. (**a**) Serum calcium concentration, adjusted for albumin concentration, revealed an increased mean serum calcium concentration in *Cdc73*^+/−^ mice with parathyroid tumours when compared to *Cdc73*^+/+^ littermates with normal parathyroids (*P<0.05). (**b**) Mean serum PTH concentration was elevated in *Cdc73*^+/−^ mice with parathyroid tumours compared to *Cdc73*^+/+^ littermates with normal parathyroids (*P<0.05). (**c**) Serum phosphate concentration in *Cdc73*^+/−^ mice with parathyroid tumours compared to *Cdc73*^+/+^ littermates with normal parathyroids. *Cdc73*^+/−^ mice with parathyroid tumours are shown as filled symbols and *Cdc73*^+/+^ littermates with normal parathyroids are shown as open symbols. Squares represent males and circles represent females. The age range of the mice was 17–24 months (mean±s.e.m.=20.0±0.30). Combined results from males and females for serum calcium, phosphate and PTH concentrations are shown, as there were no significant gender differences. Horizontal lines indicate mean values together with the standard error of the mean (s.e.m.), which is shown numerically below each group and the number (*N*) of mice.

Parathyroid tumours in the *Cdc73*^+/−^ (*n*=25, age >17 months), when compared to similarly aged wild-type *Cdc73*^+/+^ littermates (*n*=20), were associated with increased mean (±s.e.m.) serum calcium concentrations (*Cdc73*^+/−^ versus *Cdc73*^+/+^=2.85±0.04 mmol/l versus 2.66±0.08 mmol/l, *P<*0.05, Figure 4a), that was accompanied by a significantly increased mean (±s.e.m.) serum parathyroid hormone (PTH) concentration (*Cdc73*^+/−^ versus *Cdc73*^+/+^=81.34±10.29 pmol/l, versus 52.03±7.43 pmol/l, *P<*0.05, Figure 4b). The serum phosphate (Figure 4c), creatinine (data not shown) and albumin concentrations (data not shown) were not statistically different between *Cdc73*^+/−^and *Cdc73*^+/+^mice aged 17-24 months, and *Cdc73*^+/−^and *Cdc73*^+/+^mice ⩽12 months of age had no statistical differences in serum calcium, adjusted for albumin, or serum phosphate concentrations (data not shown). Thus, *Cdc73*^+/−^ mice over 17 months of age had features of primary hyperparathyroidism. *Cdc73*^+/L^/*PTH-Cre* (*n*=5, age >20 months) and *Cdc73*^*L/L*^/*PTH-Cre* (*n*=5, age >20 months) mice, when compared to *Cdc73*^+/+^/*PTH-Cre* (*n*=9) littermates also had elevated mean (±SEM) serum calcium concentrations (*Cdc73*^+/L^/*PTH-Cre*=2.81±0.07 mmol/l, *Cdc73*^*L/L*^/*PTH-Cre* 2.76±0.08 mmol/l and *Cdc73*^+/+^/*PTH-Cre*=2.52±0.07 mmol/l, *P<*0.01). The serum albumin (data not shown), creatinine (data not shown) and phosphate concentrations were not significantly different (*Cdc73*^+/L^/*PTH-Cre*=3.27±0.13 mmol/l, *Cdc73*^*L/L*^/*PTH-Cre*=3.37±0.40 mmol/l and *Cdc73*^+/+^/*PTH-Cre*=2.99±0.23 mmol/l).

### Analysis of uterine neoplasms

**Figure 5:**
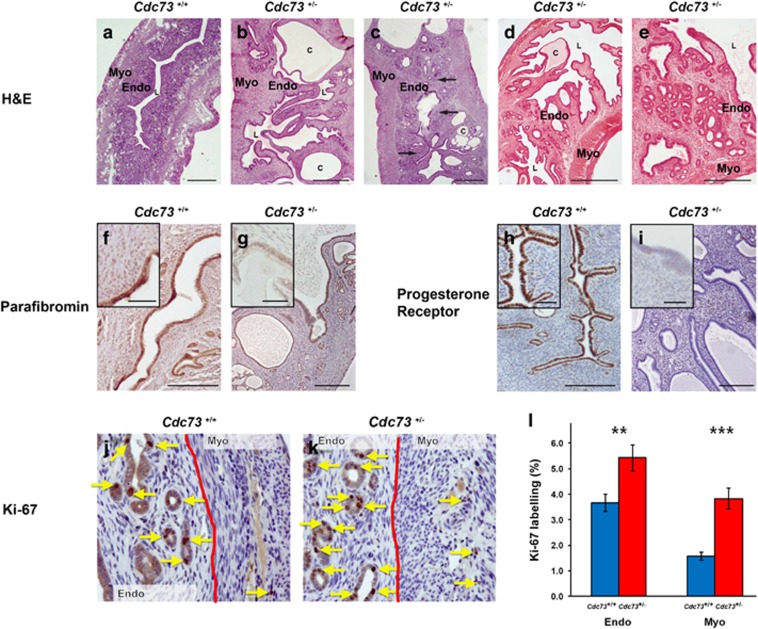
Uterine abnormalities develop in *Cdc73*^+/−^ mice. (**a**) H&E-stained section of a uterus from a *Cdc73*^+/+^ mouse showing a normal myometrium (Myo), endometrium (Endo) and central lumen (**l**). (**b**, **c**) H&E-stained sections of uteri from *Cdc73*^+/−^ mice with endometrial hyperplasia (**b**) and endometrial hyperplasia with squamous metaplasia (arrows) (**c**). Endometrial cysts (labelled **c**) and mucosal bridges traversing the lumen were observed in all the *Cdc73*^+/−^ mice with neoplasms (**b**–**e**). (**d**) H&E-stained section of a uterine adenofibroma from a *Cdc73*^+/−^ mouse with irregular polypoid endometrial projections into the lumen and cyst formation. (E) H&E-stained section of a uterine adenomyoma with glandular endometrium and irregular endometrial polyps projecting into the lumen. (**f**) Immunostaining for parafibromin in a section of a uterus from a *Cdc73*^+/+^ mouse demonstrating normal endometrial expression of parafibromin. (**g**) Parafibromin-stained sections of a uterine tumour from a *Cdc73*^+/−^ mouse demonstrating reduced parafibromin expression in the endometrium. (**h**) Section of a uterus from a *Cdc73*^+/+^ mouse demonstrating normal expression of progesterone receptor in the endometrium. (**i**) Loss of endometrial progesterone receptor expression in a uterine tumour from a *Cdc73*^+/−^ mouse. (**j**) Immunostaining for the proliferation marker Ki-67 in a section of a uterus from a *Cdc73*^+/+^ mouse (brown nuclei, arrows) with haemotoxylin nuclear counterstain. The interface of endometrium (Endo) and myometrium (Myo) is indicated by a solid red line. (**k**) Increased nuclear Ki-67 expression was observed in the endometrium and myometrium of uteri from *Cdc73*^+/−^ mice with tumours (arrows). (**l**) Quantification of Ki-67 labelling index in the endometrium and myometrium of uteri from *Cdc73*^+/+^ mice (blue bars) and from *Cdc73*^+/−^ mice with tumours (red bars) demonstrated significantly higher proliferation in *Cdc73*^+/−^ mice (**P<0.01, ***P<0.001) compared to *Cdc73*^+/+^ mice (total (*n*=) fields of view from four mice per genotype for: Cdc73^+/+^ endometrium *n*=79, Cdc73^+/−^ endometrium *n*=84, Cdc73^+/+^ myometrium *n*=78, and Cdc73^+/−^ endometrium *n*=82). All scale bars represent 100 μm; insets have 400 × magnification.

Macroscopic uterine tumours at necropsy were observed in 33.3% (*n*=8/24) of female *Cdc73*^+/−^ mice, aged ⩾18 months, but in none of 24 *Cdc73*^+/+^ littermates (*P*<0.005, two-tailed Fisher’s exact test). Histology of ⩾18 month old *Cdc73*^+/−^ mice demonstrated several abnormalities, when compared to those from *Cdc73*^+/+^ littermates (Figures 5a–e). Thus, uteri from *Cdc73*^+/+^ mice had glandular endometria with a uniform mucosal epithelium, whereas uteri from *Cdc73*^+/−^ mice had: large cysts within the endometrium (Figures 5b–e); endometrial hyperplasia with areas of squamous metaplasia (Figures 5b–c); and bridging of the endometrial lining across the uterine lumen (Figures 5b–e). Furthermore, *Cdc73*^+/−^ mice had uterine tumours which included an adenofibroma (Figure 5d) and an adenomyoma (Figure 5e), that were not observed in *Cdc73*^+/+^ littermates. Parafibromin expression was present in uteri of ⩾18 month old *Cdc73*^+/+^ mice (Figure 5f), but was reduced in uterine tumours (for example, fibroadenoma, Figure 5g) of *Cdc73*^+/−^ mice. Moreover assessment of progesterone receptor expression, which is a favourable prognostic marker in uterine tumours,^32, 33, 34, 35^ revealed strong endometrial expression of progesterone receptor in uteri of *Cdc73*^+/+^ mice (Figure 5h), but absent endometrial progesterone receptor expression in all of the hyperplastic, fibroadenoma and adenomyoma lesions from *Cdc73*^+/−^ mice (Figure 5i), which instead had increased stromal expression of progesterone receptor.

Assessment of proliferation rates, using BrdU incorporation, revealed the myometria from *Cdc73*^+/−^ mice to have a ~2-fold increase in proliferation rates when compared to that of wild-type littermates (Table 2, *P*<0.05). This was confirmed by immunostaining for Ki-67, which revealed a significantly increased proliferation in the endometria and myometria of *Cdc73*^+/−^ mice with uterine tumours, by 1.5- and 2.5-fold, respectively, when compared to equivalent uterine tissues of wild-type littermates (Figures 5j–l).

### Other tumours

Tumours of the bones, kidneys, thyroid, pancreas, or testes, which may develop in HPT-JT patients, were not detected macroscopically or microscopically in *Cdc73*^+/−^ mice, aged up to 21 months of age. Jaw bones of *Cdc73*^+/−^ mice had 2-fold increased proliferation rates compared to wild-type littermates (Table 2, *P*<0.05), but renal and pancreatic proliferation rates were similar in *Cdc73*^+/−^ and wild-type littermates (Table 2).

## Discussion

The results of our study, which established mice deleted for *Cdc73* alleles, reveal that these mice are representative of HPT-JT in man. Thus, *Cdc73*^+/−^ mice develop: parathyroid tumours in association with increased mean serum calcium concentrations and increased mean serum PTH concentrations, consistent with primary hyperparathyroidism; and uterine neoplasms, which comprised endometrial hyperplasia and cysts, adenofibroma and adenomyoma. Moreover, 75% of the parathyroid tumours were APAs, and thus these *Cdc73*^+/−^ mice with the conditional *Cdc73*^+/L^*/PTH-Cre* and *Cdc73*^*L/L*^*/PTH-Cre* mice provide important *in vivo* models for this rare but difficult to treat human neoplasm. These parathyroid tumours and uterine neoplasms had a lack (or reduction) of nuclear expresison of parafibromin, consistent with a tumour suppressor role for *Cdc73*, and similar to the findings reported in HPT-JT associated tumours in man.^36^ However, there are some differences in the frequency of tumours that develop in the *Cdc73*^+/−^ mice and patients with HPT-JT, and five of these are as follows: (1) PAs are the commonest manifestation occuring in >80% of HPT-JT patients, whereas they occurred in only 25% of *Cdc73*^+/−^ mice, the majority of which instead had APAs with features similar to PCs that occur in ~15% of HPT-JT patients (Table 1); (2) ossifying jaw fibromas occur in ~33% of HPT-JT patients, but were not found in *Cdc73*^+/−^ mice, which did however have an increased mandibular cell proliferation rate; (3) uterine adenomyosis and adenofibroma which are the commonest manifestations, occurring in 53% and 33% of women with HPT-JT respectively, were rare (or not found) in *Cdc73*^+/−^ mice, which instead had endometrial cysts that were not observed in women with HPT-JT; (4) renal hamartomas and Wilm’s tumour, which occur in >15% and <2% of HPT-JT patients, were not found in *Cdc73*^+/−^ mice; and (5) thyroid cancer, pancreatic adenocarcinoma and testicular germ cell tumours, which occur in <2% of HPT-JT patients, were not found in *Cdc73*^+/−^ mice. The basis of these inter-species differences remain to be defined but they may be partly due to: the methods of detection, which may have missed detecting the small tumours in the *Cdc73*^+/−^ mice; the possible later onset of tumours, as suggested by the finding of the increased mandibular cell proliferation rate which may represent a pre-malignant or early neoplastic phase of tumourigenesis; the possible functional redundancy of parafibromin for tumourigenesis in kidneys, pancreas, testes and jaw of mice; and the effects of species-specific genetic modifiers that might alter the phenotypic expression of the Cdc73 mutation in a species. However, it is important to note that the *Cdc73*^+/−^ mice developed two of the most common tumours, namely parathyroid tumours and uterine neoplasms that are observed in HPT-JT patients. Thus, *Cdc73*^+/−^ mice provide an *in vivo* model for the study of APAs and uterine neoplasms. The development of these tumours resulted in *Cdc73*^+/−^ mice having a reduced survival (Figure 2a). Moreover, survival of *Cdc73*^+/−^ males was significantly less than *Cdc73*^+/−^ females (Figure 2b), even though there were no differences in mean serum calcium or PTH concentrations, or the development of parathyroid tumours, between the genders. The basis of the decreased survival in *Cdc73*^+/−^ males remains unknown, but a reduction in signalling via insulin-like growth factor-1 (IGF-1), which is reported to favour female longevity in mice,^37, 38^ may be contributing, especially as parafibromin in murine embryonic fibroblasts has been reported to bind the IGF-1 promoter, and the loss of parafibromin has been observed to decrease expression of *Igf-1.*^19^ Finally, our results, which demonstrate that both conventional (*Cdc73*^+/−^) and conditional (Cdc73^+/L^/*PTH-Cre* and *Cdc73*^*L/L*^/*PTH-Cre*) knockout mice develop parathyroid tumours, indicate that *Cdc73* has a critical role in parathyroid tumourigenesis.

PCs, which have an incidence from 0.5% to 5% of primary hyperparathyroidism cases, may metastasize to regional lymph nodes or distant sites such as lungs, liver, bone or pancreas, and patients will generally die from complications of the associated hypercalcaemia.^39^ The only curative treatment for PC is en bloc resection of the primary tumour.^3^ However, PC cannot be easily distinguished from APA or PA pre-operatively or intra-operatively, in the absence of macroscopic tumour invasion or metastasis, and thus most patients with PC do not receive curative surgery and require long-term medical management.^39^ Medical therapies, including chemotherapy and radiotherapy, are ineffective with the exception of cinacalcet, an allosteric modulator of the calcium-sensing receptor, which is effective in correcting the hypercalcaemia.^39^ Thus, improved medical therapies for PC are required. Parafibromin immunostaining may represent an important prognostic marker, as loss of parafibromin immunostaining has been reported to be associated with decreased disease-free survival and tumour recurrence in patients with PCs.^40, 41^ Moreover, APAs with loss of parafibromin immunostaining are considered tumours of uncertain malignant potential as their recurrence rate is higher at 20% when compared to a 0% recurrence rate in APAs that express parafibromin.^40, 41^ Thus, our conventional and conditional *Cdc73* knockout mouse models that develop APAs lacking parafibromin expression will facilitate studies aimed at understanding the molecular pathogenesis of APAs, PCs and PAs, and in providing pre-clinical models for evaluating drugs.

*CDC73* mutations occur in ~70% of patients with sporadic, non-syndromic PCs, and in >75% of patients with HPT-JT. Indeed, *CDC73* abnormalities, either due to mutations or LOH are the major driver for PCs in humans, although expression of a mutated parafibromin protein rather than complete loss of parafibromin expression has also been reported in some PCs.^2^ In addition copy number gain of mutant *CDC73* alleles, with loss of the wild-type *CDC73* allele through focal deletion or loss of the chromosomal arm have also been reported,^42^ and the roles and mechanims of such selections of mutated *CDC73* alleles in parathyroid tumourigenesis remains to be elucidated. Mutations involving other genes are rare, and to date 6 multiple endocrine neoplasia type 1 (*MEN1*) germline mutations and 2 rearranged during transfection (*RET*) germline mutations have been reported in patients with PCs occurring in association with MEN1 and MEN2A, respectively;^43, 44, 45^ and 5 Prune Homolog 2 (*PRUNE2*) mutations (1 germline and 4 somatic) have been reported in PCs.^42^ Other genetic abnormalities that have been detected in human PCs include: retinoblastoma (*RB*) loss of heterozygocity (LOH) and loss of expression (LOE) in >85% of PCs;^46^ cyclin D1 (*CCND1*) overexpression in >90% of PCs;^30^ adenomatous polyposis coli (*APC*) LOH and LOE in ~75% of PCs;^47^ tumour protein 53 (*TP53*) LOH and LOE in 33% of PCs;^48^ glycogen synthase kinase 3-β (*GSK3β*) LOE in 33% of PCs;^47^ and enhancer of zeste homolog 2 (*EZH2*) gene amplification in 60% of PCs.^49^ Abnormalities of these genes are not necessarily associated with PCs in mice. For example *Men1*^+/−^ mice develop PAs but not carcinomas;^50^ transgenic mice overexpressing cyclin D1 develop adenomas but not carcinomas;^51^ *Rb*^+/−^ mice develop medullary thyroid carcinomas and pituitary adenocarcinomas but not PCs;^52^ and *Men1*^+/−^*/Rb*^+/−^ mice developed pituitary, thyroid and pancreatic islet hyperplasia, but not PCs.^52^ These findings indicate that loss of RB expression and increase of cyclin D1 expression may not be required for PC development in the mouse, and are consistent with our observations that RB and cyclin D1 expression were not altered in the APAs of *Cdc73*^+/−^ mice. Moreover, these finding indicate that *Cdc73* abnormalities represent the major driver for PCs in humans and APAs in mice.

Uterine corpus tumours, are common, occurring in >30% of women >40 years, and may be benign or malignant.^53^ Uterine tumours may originate from: the epithelial layer for example, endometrial hyperplasia or carcinoma; the mesenchymal layers, for example, leiomyomas (uterine fibroids), which are benign smooth muscle tumours that develop in the myometrium; or both (that is, mixed epithelial and mesenchymal) layers, for example, carcinosarcomas which have malignant epithelial and mesenchymal components, and adenosarcomas which are neoplasms composed of benign epithelium but malignant stroma.^54, 55, 56, 57^ The uterine tumours that develop in women with HPT-JT include benign tumours such as endometrial hyperplasia, adenomyosis, adenofibromas and leiomyosis, and malignant tumours, such as adenosarcomas.^13, 14^ *Cdc73*^+/−^ female mice devloped uterine tumours, that were representative of those in women with HPT-JT and these included endometrial hyperplasia, adenomyoma and adenofibroma. These uterine neoplasms developed in ~33% of female *Cdc73*^+/−^ mice (Figure 5), whilst spontaneous uterine lesions were not observed in wild-type mice in our study and are also reported to be exceedingly rare in normal wild-type mice.^58^ Thus, these *Cdc73*^+/−^ female mice provide a model to investigate the molecular basis of uterine tumourigenesis. A previous study of human uterine tumorigenesis has reported >70% of Mullerian adenosarcomas to have: copy number gain for MYB proto-oncogene like 1 (*MYBL1*), mouse double minute 2 proto-oncogene (*MDM2*) and cyclin dependent kinase 4 (*CDK4*); copy number loss for cyclin dependent kinase inhibitor 2A (*CDKN2A*), breast cancer type 1 susceptibility protein (BRCA1)-associated protein 1 (*BAP1*) and *RB1*; single nucleotide variations including nonsense mutations for *TP53* and alpha thalassaemia/mental retardation syndrome X-linked (*ATRX*); and mutations in signalling pathways notably PI3K-AKT/PTEN.^59^ In addition, >90% of leiomyomas (fibroids) have upregulation of G-protein coupled receptor 10 (*GPR10*) resulting in activation of the PI3K/AKT-mTOR pathway;^54^ while 70% of leiomyomas have a mutation of the mediator complex subunit (*MED12*) gene^60^ that encodes a scaffold protein which interacts with proteins that include *β*-catenin. It is interesting to note that parafibromin also directly interacts with *β*-catenin in the PAF complex to mediate Wnt signalling,^24^ whose dysregulation has been reported to be associated with development of intestinal and colon cancers, and it may be that similar pathways are involved in uterine tumourigenesis.^61, 62, 63^ Analysis of mouse embryonic fibroblasts from *Cdc73*^+/+^ and *Cdc73*^*-/-*^ mice revealed that the parafibromin/PAF complex regulated genes involved in cell growth and survival including the chromatin remodelling genes high mobility group AT-hook 1 (*Hmga1*) and 2 (*Hmga2)* to which parafibromin and PAF directly bind.^19^ Moreover, parafibromin may also act indirectly via HMGA1 which is a downstream mediator of aberrant Wnt signalling.^64^ Thus, loss of parafibromin expression in the mouse embryonic fibroblasts of *Cdc73*^*-/-*^ mice has been reported to lead to downregulation of *Hmga1.*^19^ However, this role of parafibromin in uterine tumourigenesis requires cautious extrapolation, as Hmga1 overexpression in transgenic female mice with 1-28 copies of *Hmga1a*, is associated with development of uterine tumours resembling human uterine adenosarcomas.^65, 66^ Finally, Wilms Tumour 1 protein (WT-1), which is often expressed in Mullerian adenosarcoma, has been reported to bind to the *CDC73* promoter and to repress *CDC73* expression in oral squamous cell carcinoma.^67^ The roles of these interactions of parafibromin in the aetiology of uterine tumourigenesis remain to be explored and our establishment of the *Cdc73*^+/−^ mice, which develop uterine tumours, will help to provide an important resource in these studies.

In summary, we have established a conventional *Cdc73*^+/−^ mouse, in which males and females develop PAs and APAs, and females develop uterine tumours; thus this *Cdc73*^+/−^ mouse model is representative of the human HPT-JT syndrome.^13, 14, 15^ Moreover, we have developed parathyroid-specific *Cdc73* knockout mouse models, which develop APAs and PAs. These mouse models will facilitate further *in vivo* investigations of the role of parafibromin in parathyroid and uterine tumourigenesis.

## Materials and methods

### Mouse studies

The generation of the conventional and conditional *Cdc73* knockout mouse models has been previously described.^19, 68^ Conventional *Cdc73*^+/−^ mice established using the embryonic stem cell line RRE190,^19^ were maintained on a C57BL/6 background for 10 generations to obtain congenic *Cdc73*^+/−^ mice. *Cdc73*^*L/L*^ mice^19^ were mated with parathyroid-specific Cre-expressing, PTH-Cre mice,^24^ to generate heterozygous *Cdc73*^+/L^/*PTH-Cre* mice. These *Cdc73*^+/L^/*PTH-Cre* mice were intercrossed to generate three genotypes expressing the Cre-recombinase: *Cdc73*^*L/L*^*/PTH-Cre*, *Cdc73*^+/L^/*PTH-Cre*, and *Cdc73*^+/+^/*PTH-Cre*. All mice were fed a standard diet (RM1 expanded diet, Special Diet Services Ltd., Witham, UK) and kept in accordance with national welfare guidelines and project license restrictions. Specifically, the animal studies were approved by the University of Oxford Ethical Review Committee and were licenced under the Animal (Scientific Procedures) Act 1986, issued by the United Kingdon Home Office Department (PLL 30/2914), and the Instituitional Animal Care and Use Committee of the Van Andel Research Institute.

*Cdc73*^+/+^ and *Cdc73*^+/−^ mice underwent a full post mortem at ~7 and >17–21 months of age, together with collection of blood samples for serum analysis and collecting of tissues for histological analysis. *Cdc73*^+/+^/*PTH-Cre*, *Cdc73*^+/L^/*PTH-Cre* and *Cdc73*^*L/L*^/*PTH-Cre* mice were studied at 7–12 months and ~20 months of age. Macroscopic and microscopic examinations for HPT-JT associated tumours was undertaken.

### Genotype studies

Genotypes of the *Cdc73*^+/+^ and *Cdc73*^+/−^ mice were determined by polymerase chain reaction (PCR) analysis of DNA using PCR primers (f 5′-GTCACAAA ACCAAAGCCTCTGGAACG-3′, r 5′-GTTACAAGGTCATGGATATTTCCACC-3′ and Geor 5′-CTGCAAGGCGATTAAGTTGGGTAACG-3′) to yield a wild-type band of *321* bp and a mutant band of 289 bp. Reverse transcriptase-PCR (RT-PCR), using total RNA extracted from *Cdc73*^+/+^ and *Cdc73*^+/−^ kidneys was performed using either *Cdc73*-specific primers 3 f (5′-GACCCGACCGAAAAGATCTAC-3′), 9r (5′-AGGCTGTTTTGTACGCAATGT-3′), and rev (5′-CCCAACAGTTGCGCAGCCTG AAT-3′) to yield a wild-type band of 593 bp, or a mutant band of 500 bp, (Figure 1b), as described.^69^ Genotypes of *Cdc73*^*L/L*^*/PTH-Cre*, *Cdc73*^+/L^/*PTH-Cre*, and *Cdc73*^+/+^/*PTH-Cre* mice were determined by PCR analysis of DNA using primers to detect the presence of LoxP and Cre-recombinase sites as previously described.^19, 24^

### Western blot analysis

Western blot analysis using total protein extracted from tissues of *Cdc73*^+/+^ and *Cdc73*^+/−^ mice was performed (Figure 1c), as previously described.^69^ The ability of the anti-parafibromin antibody to detect parafibromin was validated using siRNA targeting CDC73 (Dharmacon, Amersham, UK).

### Histology and immunohistochemistry

Tissues were fixed overnight in neutral buffered 4% paraformaldehyde before embedding and sectioning for immunohistochemical analysis. Haematoxylin and eosin staining was performed, using previously described methods.^70^ Commercially available antibodies were obtained and used according to the manufacturer’s instructions (rabbit anti-parafibromin A300-171A and anti-parafibromin IHC-00379 (Bethyl, Montgomery, TX, USA), rabbit anti-galectin-3 ab53082 (Abcam, Cambridge, UK), rat anti-Ki-67 M7249 (Dako, Glostrup, Denmark), rabbit anti-cyclin D1 clone SP4 (Thermo, Waltham, MA, USA), and rabbit anti-retinoblastoma sc-7905 (Santa Cruz, Heidelberg, Germany)). Colour reaction was developed using secondary goat anti-rabbit antibody conjugated with horseradish peroxidase (HRP) (Dako, Glostrup, Denmark) or biotinylated rabbit anti-rat antibody and streptavidin/HRP (DakoCytomation, Glostrup, Denmark) and 3,3'-diaminobenzidine chromogen (DAB) solution (Vectashield, Peterborough, UK), and nuclei were counterstained with haematoxylin, as previously described.^70^ Masson’s trichrome staining was used to assess for collagen in parathyroid tissue, such that collagen fibres were stained blue, nuclei were stained black and muscle, cytoplasm or keratin background was stained red/purple. Proliferation analysis was performed, as described,^31^ using continuous long-term administration of 1 mg/ml BrdU in drinking water, which specifically incorporates into the DNA of dividing cells, and BrdU was visualized utilizing commercially available antibodies (sheep anti-BrdU ab2285 (Abcam), Cy3-conjugated donkey anti-sheep (Jackson, West Grove, PA, USA)), as previously reported.^31^ The proportion of BrdU and Ki-67 containing nuclei was calculated from a minimum of six slides per specimen and four animals per group. Daily proliferation rates were expressed as the percentage of BrdU-containing nuclei divided by the number of days of BrdU exposure, as described.^31^ The Ki-67 proliferation index was calculated by dividing the number of Ki-67 labelled nuclei by the total number of nuclei, multiplied by 100, in randomly selected fields of view (200x magnification). ApopTag *in situ* apoptosis detection kit S7110 (Millipore, Billerica, MA, USA) was used according to manufacturer’s instructions to assess for apoptotic cells utilizing terminal deoxynucleotidyl transferase (TdT) for detection of free 3′OH DNA termini formed by DNA fragmentation, as described.^31^

Established criteria from the World Health Organization and other reports,^71, 72, 73^ were used to distinguish between PCs, APAs and PAs. Histologically, the diagnosis of PC requires demonstration of either capsular, vascular, and/or perineural tumour invasion, tumour growth into adjacent tissues, lymph node metastasis, local recurrence or distant metastasis, while APAs have features of PCs that lack unequivocal evidence for invasive growth.^71, 74^ Moreover, the presence of ⩾4 associated features of malignancy that include: capsular invasion without extension to surrounding soft tissue; mitosis >5/10 high power fields; broad intratumoural fibrous bands; coagulative tumour necrosis; diffuse sheet-like monotonous small cells with high nucleus:cytoplasmic ratio; diffuse cellular atypia; and presence of macronuclei in many tumour cells, qualifies for a diagnosis of PC, whereas the presence of only 1–3 of these features, qualifies for a diagnosis of APA.^71, 72, 73^ PAs, which are benign neoplasms, do not have any of the features and also do not have evidence of invasive growth.

### Clinical chemistry

Serum was analysed for calcium, phosphate, creatinine and albumin, as previously described.^50^ Total serum calcium (Ca^2+^) was adjusted (ACa) for albumin (Alb) using the formula: ACa=Ca^2+^ (mmol/l)−((Alb (g/l)−30) × 0.017), as described.^50^ Serum PTH was assayed using a commercial ELISA kit.^50^ Conversion to yield SI units was as follows: Ca^2+^ (mmol/l)=(serum calcium in mg/dl) × 0.2495 and PO^2−^ (mmol/l)=(serum phosphate in mg/dl) × 0.3229.

### Statistical analysis

Normally distributed data were analysed by Student’s *t*-test or ANOVA followed by Tukey’s multiple comparison post-hoc test. A two-tailed Fisher’s exact test was used for 2x2 contingency tables, and Kaplan–Meyer analysis was performed using a two-tailed Log-rank test.^50^ *P*-values <0.05 were considered statistically significant. Sample sizes are stated in the results section and the figure legends. The sample size for the survival study was selected based on a power of 80% to detect a 5% significance level (two-tailed) using equal numbers per group and a hazard ratio of 0.5; no animals were excluded, randomization was not required and blinding was not performed.

## Figures and Tables

**Table 1:** Proportion of tumours (percent) in patients with the hyperparathyroidism-jaw tumour (HPT-JT) syndrome and *Cdc73* ^*+/−*^ mice

*Tumour*	*HPT-JT patients*	*Cdc73*^+/−^ *mice (⩾18 months)*
Parathyroid	82% adenoma (108/132)^1^ >15% carcinoma (20/132)^1, 9^	68% overall 25% adenoma 75% atypical adenoma
Mandible	33% ossifying fibroma (67/205)^9^	0%
Uterus	74% overall (20/27)^7, 9^ 53% adenomyosis (8/15) 33% adenofibroma (5/15) 27% endometrial hyperplasia (4/15) 27% leiomyoma (4/15) 13% adenosarcoma (2/15)	33% overall 100% endometrial cysts 25% endometrial hyperplasia 13% adenofibroma 13% adenomyoma
Kidney	16% hamartoma (21/132)^5^ <2% Wilms' tumour (3)^4^	0%
Thyroid	<2% Papillary thyroid carcinoma (2)^7^ <1% Hurthle cell adenoma (1)^8^	0%
Pancreas	<1% Adenocarcinoma (1)^8^	0%
Testis	<1% Mixed germ cell tumour (1)^8^	0%

**Table 2:** Daily proliferation rates of conditional and conventional *Cdc73* knockout mice, assessed by incorporation of BrdU, in parathyroid, jawbone, kidney, pancreas and uterine tissues

*Tissue*	*Genotype*	*Mean proliferation rate*^a^	*Fold change*	P*-value*
		*%/day±s.e.m.*	vs *wild type*	vs *Cdc73*^+/+^
*Parathyroid*				
	*Cdc73*^+/+^	0.150±0.020	—	—
	*Cdc73*^+/*−*^	0.628±0.101	4.2	0.0001
	*Cdc73*^+/+^/*PTH-Cre*	0.165±0.016	—	0.380
	*Cdc73*^+/L^/*PTH-Cre*	0.513±0.053	3.1	<0.0001
	*Cdc73*^L/L^/*PTH-Cre*	1.416±0.389	8.6	<0.0001
*Mandible*				
	*Cdc73*^+/+^	0.653±0.170	—	—
	*Cdc73*^*+/*^*^−^*	1.476±0.254	2.3	0.014
*Uterus*				
Myometrium	*Cdc73*^+/+^	0.526±0.063	—	—
	*Cdc73*^+/−^	0.900±0.168	1.7	0.046
Endometrium	*Cdc73*^+/+^	1.903±0.244	—	—
	*Cdc73*^+/−^	1.932±0.175	1.0	0.924
*Kidney*				
	*Cdc73*^+/+^	0.371±0.023	—	—
	*Cdc73*^+/−^	0.374±0.023	1.0	0.925
*Pancreas*				
Exocrine	*Cdc73*^+/+^	0.208±0.032	—	—
	*Cdc73*^+/−^	0.293±0.046	1.4	0.136
Endocrine	*Cdc73*^+/+^	0.363±0.045	—	—
	*Cdc73*^+/−^	0.398±0.033	1.1	0.533

Abbreviation: *cdc73*, cell division cycle 73.

a A minimum of four animals per genotype and a minimum of four sections per animal were analysed.
