# Supplementary material for: Mice deleted for cell division cycle 73 gene develop parathyroid and uterine tumours: model for the hyperparathyroidism-jaw tumour syndrome
Source: Oncogene. 2017 Mar 13;36(28):4025–36. doi: 10.1038/onc.2017.43 (PMC5472200; doi:10.1038/onc.2017.43)
Supplement: Supplementary Information [file onc201743x1.doc]

Supplementary Figure 1. Validation of anti-parafibromin antibody in siRNA-transfected HeLa cells

HeLa cells were transfected with 40nM siRNA targeting *CDC73* (siCDC73) or non-targeting control (siNT) and harvested 72h later. (A) Western blot analysis utilising an anti-parafibromin antibody (A300-171A) revealed expression of parafibromin protein (64kD) in siNT-treated cells which was reduced in cells treated with siCDC73. (B) Analysis of band intensity normalised for alpha-tubulin expression showed parafibromin expression in siCDC73-treated cells was ~55% of that observed in siNT-treated cells confirming the ability of the antibody to detect parafibromin. ***p*<0.01, n=4. Several higher molecular weight bands were also detected, however, expression of these was unaffected by treatment with siCDC73 suggesting non-specific antibody interactions. In addition, the results from HeLa cells which reveal non-specific bands that are different to those from the kidney (Figure 1D) indicate that these can vary in different tissues.

Supplementary Figure 2. Increase in mean length of parathyroid glands in mutant mice

A) Mean length of parathyroid glands in *Cdc73^+/+^* and parathyroid tumours in *Cdc73^+/-^* mice. ***P*<0.01. B) Mean length of parathyroid glands in *Cdc73^+/+^/PTH-Cre* and parathyroid tumours in *Cdc73^+/L^/PTH-Cre* and *Cdc73^L/L^/PTH-Cre* mice. **P*<0.05. Horizontal lines indicate the means with standard error of the mean (SEM).

Supplementary Figure 3. Parafibromin expression in non-parathyroid tissues of *Cdc73^+/+^/PTH-Cre*, *Cdc73^+/L^/PTH-Cre* and *Cdc73^L/L^/PTH-Cre* mice

Nuclear parafibromin protein expression (brown) in pancreases and thryoids was similar in *Cdc73^+/L^/PTH-Cre* and *Cdc73^L/L^/PTH-Cre* mice when compared to *Cdc73^+/+^/PTH-Cre* mice. Parafibromin expression is observed: in pancreatic exocrine (large arrows) and endocrine (islet) cells (small arrows); in endothelial cells lining blood vessels of the pancreas (medium arrow); and flattened epithelial cells lining the follicles of the thyroid (broad arrows). These results indicate that the loss of parafibromin expression is parathyroid-specific in the *Cdc73^+/L^/PTH-Cre* and *Cdc73^L/L^/PTH-Cre* mice. B) Nuclear parafibromin protein expression (brown) in mucosal cells. Scale bars represent 100μm.

Supplementary Figure 4. Apoptosis rates by TUNEL assay in parathyroid tumours from *Cdc73^+/+^* *Cdc73^+/-^*, *Cdc73^+/+^/PTH-Cre*, *Cdc73^+/L^/PTH-Cre* and *Cdc73^L/L^/PTH-Cre* mice. WT includes *Cdc73^+/+^* (n = 5) and *Cdc73^+/+^/PTH-Cre* (n = 6) mice. Horizontal lines indicate the means ± SEM. ****P*<0.005. The apoptotic rates in parathyroids of *Cdc73^+/-^*, *Cdc73^+/L^*/*PTH-Cre* and *Cdc73^L/L^/PTH-Cre* mice were not significantly different from wild-type (*Cdc73^+/+^* and *Cdc73^+/+^/PTH-Cre* littermates), although the apoptotic rate in *Cdc73^L/L^/PTH-Cre* was significantly higher than that in *Cdc73^+/L^/PTH-Cre* mice (*P*<0.005), which may partially explain the lack of significant enlargement of the parathyroid glands in *Cdc73^L/L^/PTH-Cre* mice compared to *Cdc73^+/+^/PTH-Cre* mice (Supplementary Figure 2).
